# From encoding to remembering: pragmatic inferences reveal distinct routes of word learning in autistic children

**DOI:** 10.3389/fnhum.2025.1633013

**Published:** 2025-08-21

**Authors:** Katherine Marie Trice, Zhenghan Qi

**Affiliations:** ^1^Department of Psychology, Northeastern University, Boston, MA, United States; ^2^Department of Communication Sciences and Disorders, Northeastern University, Boston, MA, United States

**Keywords:** autism, mentalizing, pragmatics, word learning, individual differences, eye-tracking

## Abstract

Mentalizing skills—the capacity to attribute mental states—play critical roles in word learning during typical language development. In autism, mentalizing difficulties may constrain word-learning pathways, limiting language-acquisition opportunities. We ask how autistic children encode and retrieve novel words and what drives individual differences. We test whether autistic children’s word learning benefits from pragmatic inferences, as in non-autistic. Forty-nine 6-to-9-year-old verbal autistic children participated. During learning, four novel words in the direct-mapping condition (DM) could be uniquely mapped to one novel object and four in the pragmatic-inference condition (PI) required children to assume speaker intent. Immediate recall and retention (15-min delay) were tested via four-alternative-forced-choice-task. Autistic children showed above-chance PI mapping, no immediate recall differences, and PI retention advantage. However, individual difference analyses suggest a bimodal PI-retention pattern: 55% showed above-chance PI word recognition (PI-Retained) and 45% at-or-below-chance (PI-Limited). Retention profiles do not reflect general memory—most PI-Limited children remembered DM words well. Instead, profile was associated directly with learning success. For PI-Limited specifically, learning performance was at-chance. Eye-movement during learning showed converging evidence: only PI-Retained consistently diverged between looks-to-target and competitor. Only nonverbal IQ in conjunction with initial mapping reliably differentiated groups, not mentalizing or language measures. This suggests distinct pathways of word-meaning acquisition in autistic children with otherwise similar profiles. While PI resolution may facilitate word-meaning acquisition for some, DM better serves others. This underscores the importance of characterizing learning processes as a pathway to understanding the heterogeneity of language in autism.

## 1 Introduction

Children frequently encounter novel words in a complex world. To narrow down possible meanings, they often must weigh multiple linguistic and extralinguistic cues to resolve potential ambiguity and assign the correct label-to-referent mapping (Fisher et al., 2010; Gleitman, 1990; Halberda, 2003, 2006; Naigles, 1990). Weighting of these cues changes over development as mentalizing skills mature and more accurate judgment and leveraging of other’s communicative intent is seen (Robinson and Whittaker, 1985; Frank and Goodman, 2014; Katsos and Bishop, 2011; Grigoroglou and Papafragou, 2019). Take, for example, the inference of mutual exclusivity – where one maps a novel label to a novel over known object. Typically, in such instances, nothing about the language itself cues the child which the novel word is. Instead, they must infer that the speaker intends the novel object, as if they had intended the known object, they likely would have utilized the known label. Such inferencing abilities have been tied to mentalizing (e.g., the skills underlying computing, understanding, and applying beliefs, desires, perspectives, intentions of others; Diesendruck and Markson, 2001; cf. Grice, 1975; Sperber and Wilson, 1986). Success at other pragmatic phenomena – from perspective-taking to irony comprehension to navigating speaker knowledge – have been similarly linked (Fairchild and Papafragou, 2021; Matthews et al., 2018; Brosseau-Liard et al., 2015; Sabbagh and Baldwin, 2001). Furthermore, within word-learning, more complex pragmatic inferences – for instance, when speaker intent must be used to distinguish between novel objects or assign a new label to known – can similarly be reliably resolved by children to map words and have been likewise linked in memory to mentalizing (Frank and Goodman, 2014; Trice et al., 2025).

However, this link between mentalizing and word learning raises a critical question—what about autistic children? Difficulties related to mentalizing abilities are central aspects of autism, and autistic individuals face mentalizing challenges across their lifespan (American Psychiatric Association, 2013; Baron-Cohen et al., 1985; Senju, 2012; Rosenthal et al., 2019). Prior word learning research has often focused on social cue attention, like eye gaze and pointing, or the use of relatively basic inferences, like mutual exclusivity (Tenenbaum et al., 2014; Gliga et al., 2012; Carpenter et al., 2002; Preissler and Carey, 2005; de Marchena et al., 2011). Such abilities emerge in typically-developing children in early infancy up through their second year (Carpenter et al., 1998; Morales et al., 1998; Halberda, 2003; Bion et al., 2013). Thus, even when success at mutual exclusivity is seen in autistic children, it can be difficult to determine whether it is likely to hold for more complex pragmatic inferences with more sophisticated computations. And if so, whether the inferences made are underpinned by mentalizing in autism or alternative compensatory routes.

The possibility of either is made particularly salient by work on mentalizing in autistic individuals with typical-like verbal abilities. Here, research on mentalizing ability– including how it interfaces with pragmatics—has demonstrated a mix of delays in development, possible compensatory mechanisms supporting behavioral success (especially in explicit metalizing tasks), and difficulties only with specific aspects of mentalizing rather than the whole (Williams et al., 2013; Colich et al., 2012; Senju, 2012; Rosenthal et al., 2019). As such, delays, difficulties, and even differences in the mechanism used are all possibilities for pragmatic inference resolution, and in turn, the word-learning pathways most beneficial for a given autistic individual to navigate vocabulary acquisition in ambiguity. Given the heterogeneity of profiles in autism, this is true not only for those who struggle visibly with language acquisition, but in considering the routes different autistic individuals may take to achieve broad linguistic success.

Furthermore, work on how mentalizing influences word retention beyond in-the-moment mapping is limited. This as a point of concern, as successful initial mapping does not always transition into long-term retention (Axelsson et al., 2012; Horst and Samuelson, 2008; Bredemann and Vlach, 2021). This gap in the literature intersects with a major concern in autism research: the heterogeneity of language in autism. While language impairment is no longer a diagnostic criterion, we still see up to 60% of autistic individuals present with co-occurring language impairments, including vocabulary; similar to mentalizing difficulties, such impairments often do not resolve with age even for those without other significant cognitive disabilities (Durrleman et al., 2015; Colle et al., 2008; Lewis et al., 2008). Language skills, and vocabulary in particular, strongly predict autistic individual’s long-term vocational, educational, and social success (Tager-Flusberg et al., 2011). Identifying potential word-learning scenarios that hinder or facilitate vocabulary growth offers promising targets for language intervention, especially for early school years, as the average age of autism diagnosis is not until age 5 (Van’T Hof et al., 2021). Compared to early childhood, the learning environment in the classrooms offer fewer opportunities for word learning through unambiguous one-on-one instructions due to other targeted educational and social goals. With vocabulary size for typical individuals increasing from approximately 10,000 to up to 40,000 between the ages of 5 and 10, strategies to ensure appropriate growth for autistic children are paramount (Shipley and McAfee, 2015; Anglin, 1993).

In our prior work, typically-developing children as young as 4 could accurately resolve complex pragmatic inferences. A memory advantage for pragmatically-inferred words over those unambiguously mapped began emerging by 6 (Trice et al., 2025). However, with the strong link to mentalizing abilities, whether complex pragmatic inferences serve as an opportunity or a barrier for autistic children remains unclear. This study thus seeks to determine if school-aged autistic children (6-9-year-olds) can reliably map words in pragmatic-inferential contexts, and if so, whether they see a memory advantage. It will examine the profile of autistic individuals who show – or fail to show – such an advantage across domains such as mentalizing, language, and cognition.

## 2 Materials and methods

### 2.1 Participants

49 children, recruited via Simons Powering Autism Research (SPARK)’s database, were included. All were 6;0–8;11-years-old, professionally diagnosed with autism, native English speakers with no extensive exposure (>10 h/week) to other languages before age 5, able to speak at least three-word sentences, and if they scored below the autistic threshold on the Social Communication Questionnaire, did not have their diagnosis in-question in SPARK. This surpassed the 80% power threshold based on neurotypical children (Trice et al., 2025). 18 participants did not complete all additional measures, for a total of 9% of the individual difference data missing. All parents/guardians gave informed consent and children assent. Basic participant demographics are in Table 1. Further demographics—including diagnoses and intervention services—are in Supplementary Table A1 and Supplementary Figure A1.

**Table 1:** Basic demographics.

Demographic	Mean	SD	Range	Count	%
Age	7;7	0;11	6;0-8;10		
Age at ASD Diagnosis (Months)*	41.6	12.9	12-68		
Gender					
Girl				14	29%
Boy				35	71%
Race/Ethnicity					
White				31	58%
Asian				3	6%
Black or African American				8	15%
Hispanic or Latino				5	9%
American Indian or Alaska Native				1	2%
Not Reported				1	2%
Area Deprivation Index	36.1	23.9	1-98		
Social Communication Questionnaire	18	6.3	5-30		
ABI-S	6.2	2.1	2-9.4		
KBIT (Non-Verbal, Standard Score)	108.1	27.3	41-147		
PVT (Standard Score)	103.4	18.5	71-146		
ORR (Standard Score)	106.5	16.3	80-132		
RSR (Standard Score)	87.5	23.0	40-118		
ToM Booklet Score**	0.65	0.21	0.19-0.92		
MitE Score**	8.4	2.4	2-12		

*Provided by SPARK.

**As the ToM Booklet Task, modified from Richardson et al. (2018), and the MitE task (child version, Pahnke et al., 2020), are not broadly-used assessments, the setup of each is further discussed in Supplementary Materials Section 3.

ASD, Autism Spectrum Disorder; ABI-S, Autism Behavioral Inventory – Short (Bangerter et al., 2017); KBIT, Kaufman Brief Intelligence Test-II; PVT, National Institute of Health Toolbox Picture Vocabulary Test (Gershon et al., 2014b); ORR, National Institute of Health Toolbox Oral Reading Recognition Test (Gershon et al., 2014a); RSR, Redmond Sentence Recall task (Redmond, 2007); ToM, Theory of Mind; MITE, Mind in the Eyes Task standardized for children (Pahnke et al., 2020).

### 2.2 Stimuli and procedure

The experiment was conducted via Zoom. The main task [identical to Trice et al. (2025)] was divided into the following phases: word-learning, including immediate recall of just-learned words (6 min); 15-min break (where a mentalizing task was completed); retention of all learned words (1 min). To limit fatigue, individual difference tasks were split over two sessions. The word learning experiment was hosted on Gorilla Experimental Builder (Anwyl-Irvine et al., 2020).

#### 2.2.1 Word learning task

**Figure 1:**
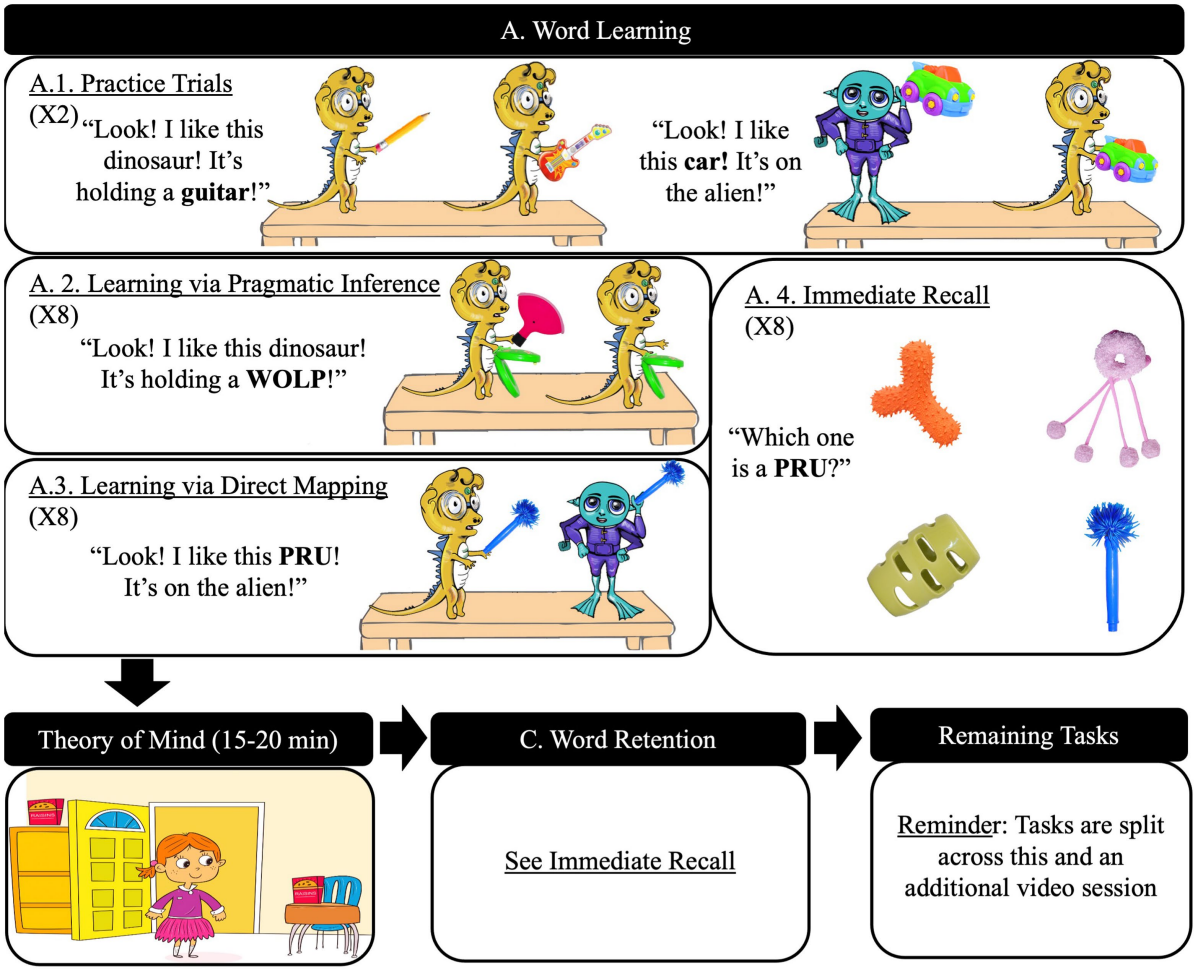
Experimental procedure and stimuli example. This figure demonstrates example trials for introduction, word learning, immediate recall tasks and retention tasks, along with the task order. Text in quotations would be spoken aloud. Adapted from Trice et al. (2025) The Unforgettable “Mel”: Pragmatic Inferences Affect How Children Acquire and Remember Word Meanings published by John Wiley & Sons Ltd. (licensed under CC BY-NC 4.0 https://creativecommons.org/licenses/by-nc/4.0/. Modifications are the replacement of stimuli in panels A.2 and A.3 with those referenced in the current text; the rephrasing of the figure caption, and replacement of panel D. Executive Functioning with a new panel on the remaining tasks in this paper).

Children learned nonce words within two conditions, Pragmatic Inference (PI) and Direct Mapping (DM), within-subject (Figure 1).

Before word learning, children were introduced to a virtual toy store and character. Children were instructed to select the toy the character wants and practiced with known objects (Figure 1A.1). This allowed for familiarization of dinosaur and alien figures. Feedback was given.

For PI, the correct referent could be determined via pragmatic inference: assuming a speaker intends their utterance to be clear and informative and believes that they have given listeners precisely enough information to accomplish this. For example, as in Figure 1A.2, children would hear “Look! I want this dinosaur! It’s holding a ‘wolp’!” then be asked to select the wanted toy. They would see two identical dinosaur figures, both holding an identical novel object (e.g., the green clam-shaped novel objects), and one holding an additional unique novel object (e.g., the red fan-shaped novel object). To identify the correct dinosaur, the listener must infer that the speaker intends for the novel referent to disambiguate the dinosaurs and believes that they have given enough information to do so. Therefore, under this informativity assumption, ‘wolp’ must refer to the unique novel object that is the disambiguating feature. Thus, a language learner can map ‘wolp’ to the red object, learning a new word. Children’s success in choosing the right referent was used as a measure of in-the-moment mapping.

For DM, the referent could be determined based purely on the visual and linguistic setup without complex inferencing. For example, as in Figure 1A.3, a child would hear “Look! I want this ‘pru’! It’s on the alien!” then be asked to select the wanted toy. The child would see a dinosaur and an alien figure, each holding a single identical novel object (e.g., the blue pen-shaped novel objects). As there are no other novel objects present, and the practice and filler trials have established that the target is the object held by one of the figures, this mapping is straightforward for the learner with little or no ambiguity.

Learning conditions were counterbalanced in a block design, one block per condition. Each featured four novel words, two trials per word, in randomized order. Words and objects were unique to condition. These were identical to Trice et al. (2025) and were selected from the Noun Database Project and paired at-random (Horst and Hout, 2016). Pairs were kept consistent within-condition to minimize extraneous factors in individual difference analyses. Filler trials with known words were interspersed. No feedback was given. Children’s eye-movement was recorded trial-by-trial using participants’ webcams. Experimenters were trained in directing parents and children to ensure high-quality recording (Ovans et al., 2025).

Immediate recall (Figure 1A.4) and retention (Figure 1C) both used a four-alternative-forced-choice task to test memory of newly-acquired words, with each relevant word tested once in randomized order. Children would pick the correct referent upon hearing “Which one is a [novel word].” Immediate recall would be administered after the completion of the learning block for a given condition, testing the four novel words learned for that condition. The retention phase after the 15-min break tested all eight novel words.

#### 2.2.2 Individual difference measures

##### 2.2.2.1 Behavioral measures

Individual difference measures included both parent- and child-competed assessments. These characterize children in the following domains: language, mentalizing, cognition, prior interventions, and demographics. See Table 2 for descriptions, and Supplementary Table A2 for descriptive statistics.

**Table 2:** Individual difference measures.

Category	Measure	Assesses	Justification
Language	PVT Standard Score	Vocabulary knowledge	Jointly captures linguistic abilities across domains, from phonological to lexical to syntactic to daily integration in communications, leading to a holistic language profile
	ORR Standard Score	Reading ability and phonological awareness	
	RSR Standard Score	Grammatical abilities	
	SCQ Communication Sub-Score	Caregiver-observed basic communicative skills	
Mentalizing	Selected ToM Booklet Score*	Variety of cognitive ToM constructs developing across age range	Captures ToM development, particularly when dealing with referential ambiguity
	MitE Score*	Affective mentalizing ability via visual emotional identification	Expands to broader mentalizing abilities
	ABI-S Social Communication Score	Caregiver-observed quality and frequency aspects of social interaction	Captures navigation of social understanding in daily life
Services	ASD Services**	Whether the child receives services related to their autism diagnosis	Captures impact of ongoing interventions and the need for such interventions, in terms of autism-related, education elated, and language related traits
	IEP Services**	Whether the child has an IEP, and if so, whether there is a language component	
General	Non-Verbal KBIT	Non-verbal IQ	Captures effect of general non-linguistic pattern observation/reasoning
	Gender	Child’s gender	Controls for effects of gender-based phenotypic variation and speaker affinity
	Current Age	Child’s age at time of main assessment	Assesses developmental time course
	Diagnosis Age**	Age at original autism diagnosis	Assesses impact of early or later diagnosis

*As the ToM Booklet Task, modified from Richardson et al. (2018), and the MitE task (child version, Pahnke et al., 2020), are not broadly-used assessments, the setup of each is further discussed in Supplementary Materials Section 3.

**Provided by SPARK.

PVT, National Institute of Health Toolbox Picture Vocabulary Test (Gershon et al., 2014b); ORR, National Institute of Health Toolbox Oral Reading Recognition Test (Gershon et al., 2014a); RSR, Redmond Sentence Recall task (Redmond, 2007); SCQ, Social Communication Questionnaire (Rutter et al., 2003); ToM, Theory of Mind; MITE, Mind in the Eyes Task standardized for children (Pahnke et al., 2020); ABI-S, Autism Behavioral Inventory – Short (Bangerter et al., 2017); ASD, Autism Spectrum Disorder; IEP, Individualized Education Plan; KBIT, Kaufman Brief Intelligence Test-II.

##### 2.2.2.2 Eye-tracking

Eye-gaze data during PI mapping was collected to capture the online processing of pragmatic cues. Webcam data (sampled at 30 Hz) were coded by trained researchers. For each trial in each condition, eye-gaze was segmented beginning 0.2 s before and ending 1.8 s after novel-word onset and coded frame-by-frame for fixation-to-target, to-competitor, away, or un-codable (subsequently dropped). Codable data were divided into time bins of 8 observations per bin.

### 2.3 Analysis approach

While in many cases, Pearson t-tests were deemed appropriate, sometimes more complex models/methods were utilized. Supplementary Materials Section 4 covers construction details. Where appropriate, results were compared to neurotypical peers from Trice et al. (2025). All analyses were run in R (R Core Team, 2021).

## 3 Results

### 3.1 Overall success

For initial mapping (chance = 0.5), autistic children were able to correctly resolve pragmatic inferences above-chance for PI (0.62 ± 0.05, *t* = 2.81, df = 48, *p* = 0.007, *d* = 0.40). Unsurprisingly, children performed near-ceiling for DM, as their job was to click on the novel object on the named character and thus room for error was limited (0.93 ± 0.03, *t* = 25.22, df = 48, *p* < 0.001, *d* = 3.60). Split-half reliability (*split-half,* Parsons, 2021) for PI initial mapping was 0.78.

Immediate recall (chance = 0.25) remained above-chance for both conditions (PI: 0.51 ± 0.09, *t* = 5.64, df = 48, *p* < 0.001, *d* = 0.80; DM: 0.44 ± 0.10, *t* = 3.0, df = 48, *p* < 0.001, *d* = 0.54). No effects of condition, gender, or initial mapping were seen (*p*’s > 0.10; Supplementary Table A3). Split-half reliability was 0.50 for PI and 0.64 for DM.

Retention (chance = 0.25) remained above-chance for both conditions (PI: 0.51 ± 0.09, *t* = 5.72, df = 47, *p* < 0.001, 0.83; DM: 0.43 ± 0.08, *t* = 4.37, df = 47, *p* < 0.001, 0.63)^1^. There was a memory advantage of PI over DM (*β* = 0.89, SE = 0.30, z = 2.92, *p* = 0.004, *R*^2^ = 0.02). Children with more accurate initial mapping showed greater retention (*β* = 1.06, SE = 0.30, *z* = 3.58, *p* < 0.001, *R*^2^ = 0.04). No gender effects were seen (Supplementary Table A3). Split-half reliability was 0.45 for PI and 0.48 for DM.

This pattern is similar to age-matched neurotypical peers, as PI and DM mappings are both above-chance and a retention advantage for PI over DM was present (Trice et al., 2025).

### 3.2 Sub-group behavior

As autistic individuals are markedly heterogeneous, we looked participant-by-participant at PI retention to see if a distinct pattern emerged.

**Figure 2:**
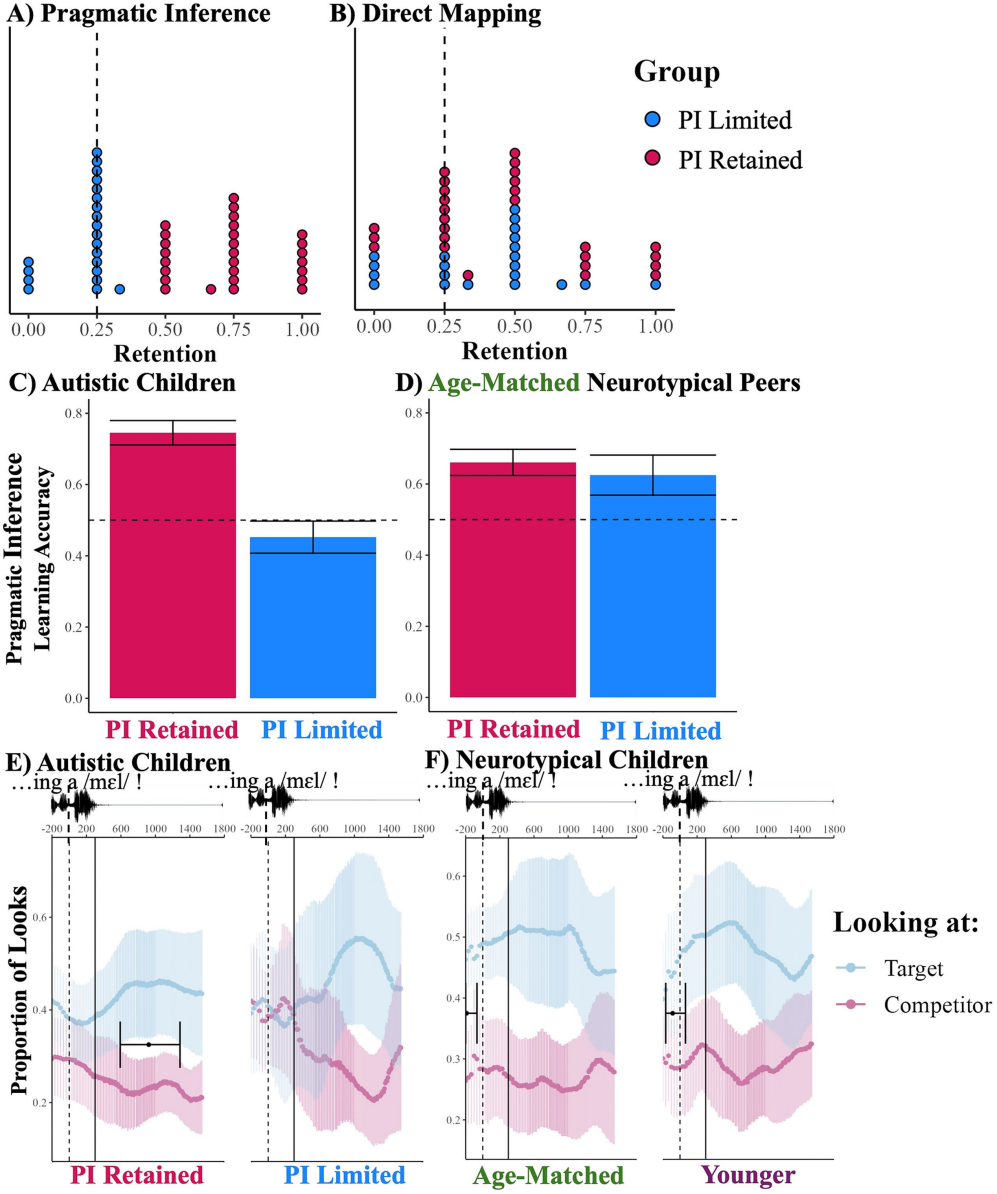
Learning and retention results by sub-group. Participants were grouped based on whether they correctly identified ≥50% of the novel words in the pragmatic inference condition during retention (PI-Retained) or correctly identified <50% (PI-Limited). **A,B** show individual retention scores for autistic children. Dots represents mean retention value for a given participant for the condition. Vertical line (0.25) represents chance. **C,D** show initial mapping of pragmatically inferred words for autistic and their age-matched neurotypical children. Bars represent mean accuracy. Error bars represent within-subject standard error. Horizontal line (0.5) represents chance. **E,F** show eye-gaze divergence during the novel word for the pragmatic inference condition learning. Time course across the top is in ms. Solid black points represent divergence point. Error bars represent 95% CI. Vertical dashed line (0) marks word onset, solid vertical line represents earliest saccade if triggered by word onset. Target and competitor proportions do not sum to one, as looks away, while not plotted here, are still factored into relative fixation at any given timepoint. Panel **F** adapted from Trice et al. (2025) The Unforgettable “Mel”: Pragmatic Inferences Affect How Children Acquire and Remember Word Meanings published by John Wiley & Sons Ltd. (licensed under CC BY-NC 4.0 https://creativecommons.org/licenses/by-nc/4.0/. Modifications are changing group name from older to age-matched).

Here, the significant between-subject variability formed a nearly-bimodal distribution, as reinforced by a two-cluster model being the best fit Gaussian mixture model (*mclust*), motivating us to study these two subgroups (Figure 2A). As the split occurs along an accuracy of 0.5, representing those that retained two out of four words in a task with 0.25-chance, we are confident that the children at 0.5 performed substantially above-chance and should be grouped with those demonstrating successful retention. Thus, our sample was divided into PI-Retained (*n* = 27, PI: 0.74 ± 0.08; DM: 0.47 ± 0.12) – those who successfully remembered the pragmatically-inferred words (accuracy≥0.50, chance = 0.25) – and PI-Limited – those who performed at- or below-chance (*n* = 21, PI: 0.21 ± 0.05; DM: 0.39 ± 0.12). Both the PI-Retained and the PI-Limited group performed similarly in the DM retention, both substantially above-chance, indicating that PI-Limited cannot be fully explained by poor memory across conditions (PI-Retained: *t* = 3.58, df = 26, *p* = 0.001, *d* = 0.69; PI-Limited: *t* = 2.50, df = 20, *p* = 0.02, *d* = 0.54; Group Comparison: *t* = 0.88, df = 46, *p* = 0.38; Figure 2B). Thus, opposite patterns of context effect were found between groups: the PI-Retained group showed a memory advantage of PI, while the PI-Limited group showed a marginal memory advantage of DM (PI-Retained: *β* = 1.65, SE = 0.42, *z* = 3.89, *p* < 0.001, *R*^2^ = 0.04; PI-Forgotten: *β* = −1.07, SE = 0.56, *z* = −1.90, *p* = 0.06, *R*^2^ = 0.01).

Next, we examined the relationship between PI initial mapping and PI retention. These timepoints correlated significantly and robustly (*r* = 0.47, *p* = 0.001). Sub-groups were statistically differentiable during initial mapping, with only PI-Retained above-chance (PI-Limited: 0.45 ± 0.13, *t* = −0.76, df = 20, *p* = 0.45; PI-Retained: 0.75 ± 0.10, *t* = 4.93, df = 26, *p* < 0.001, *d* = 0.95; Subgroup-Difference: *t* = 3.67, df = 40.76, *p* < 0.001, *d* = 1.08; Figure 2C).

This bimodal distribution is unlike the age-matched peers in Trice et al. (2025). When the neurotypical sample is split into PI-Limited (36% of sample) and PI-Retained subgroups using the same accuracy cutoff, we see no initial-mapping accuracy difference (PI-Limited: 0.63 ± 0.19; PI-Retained: 0.66 ± 0.15; Subgroup-Difference: *t* = 0.31, df = 25.44, *p* = 0.76; Figure 2D). Furthermore, in Trice et al. (2025), even for younger neurotypical children – who do not show a PI retention advantage, similar to PI-Limited—accuracy during initial mapping remained above-chance. Notably, scores in the mentalizing task related to PI in neurotypical children did not statistically differ between younger neurotypical children and the PI-Limited group (PI-Limited: 0.60 ± 0.10; Younger-Neurotypical: 0.57 ± 0.07; Group-Difference: *t* = 0.51, df = 38.84, *p* = 0.61).

The initial mapping difference between the subgroups is supported by eye-tracking. In PI-Limited, looks between target and competitor never diverged as they did for PI-Retained (PI-Limited: no divergence; PI-Retained: 921 ± 329 ms; Figure 2E). However, for PI-Retained, this divergence occurred well after any saccade triggered by the start of the novel word. The neurotypical children in Trice et al. (2025) exhibited divergence between the looks-to-target vs. competitor well before final-word onset (Figure 2F). Thus, autistic children did not preemptively resolve referential ambiguity, instead evaluating for it only after the full statement was given and continued ambiguity remained clear.

### 3.3 Sub-group individual differences

Finally, we sought to better understand the individual difference predictors of different word learning profiles.

First, we examined each individual difference measure separately (Table 2 for measures, Supplementary Table A4 for sub-group comparisons). Only the Social Communication Questionnaire Communication subscore – a metric of very basic communicative skills – emerged as a difference, with greater difficulty for PI-Limited. However, as this *p*-value was only 0.05 before multiple-comparison correction, it is not statistically robust enough to be taken as a definitive difference.

Next, we examined whether composite scores of mentalizing or language tasks were able to predict group membership (Supplementary Table A5). Language, theory of mind, and age failed to predict membership. However, PI initial mapping in conjunction with non-verbal IQ significantly and robustly predicted membership while non-verbal IQ by-itself did not (R^2^: Non-Verbal IQ = 0.03, PI Initial Mapping = 0.18, PI Initial Mapping + Non-Verbal IQ = 0.36; Combined Model: β_PI-Initial-Mapping_ = 6.17, SE_PI-Initial-Mapping_ = 1.82, z _PI-Initial-Mapping_ = 3.39, p_PI-Initial-Mapping_<0.001, β_Non-Verbal-IQ_ = 0.04, SE_Non-Verbal-IQ_ = 0.02, z_Non-Verbal-IQ_ = 2.34, p_Non-Verbal-IQ_ < 0.01).

Finally, we ran a data-driven cross-validation approach to determine if any of our other variables could tease apart our groups but did not find any combination of measures that did (Supplementary Table A6).

## 4 Discussion

Our findings present an overall PI memory advantage for autistic individuals – just like their typically-developing peers. Importantly, these data also revealed significant variation in how autistic children encode and retain novel words in contexts where pragmatic inference is necessary. About half of the autistic children in our sample correctly mapped novel words through PI and retained these words above-chance, indicating that more complex pragmatic inferences do not pose a barrier for all autistic individuals for language growth. Instead, they may serve as a tool to expand vocabulary and assist in language development in mixed settings. However, unlike neurotypical peers, these autistic children’s eye-gaze data suggest that such pragmatic computation was delayed until they encountered the novel word.

In contrast, the other half of the autistic children did not demonstrate successful retention of PI words and seemed to be unable to apply assumptions of informativity for referential disambiguation. This pattern is different from younger neurotypical children (4-6-year-olds) in Trice et al. (2025). There, even though younger children did not retain more inferred than directly-mapped words, initial mapping success was evident.

Finally, no individual difference measures distinguished the two sub-groups, who otherwise presented robust and reliable distinction in their immediate mapping and retention behavior during PI word learning. Only the combination of immediate mapping and non-verbal IQ reliably predicted who has the potential to benefit from mapping words via PI. In the absence of clean-cut profile explanations that align with a theoretical framework, we must turn our attention back to the stages of word learning themselves.

For initial mapping, this variation may stem from differences in weighting of relevant cues. The lack of subgroup difference in mentalizing ability may indicate that this alone does not determine autistic children’s success in this word learning task. Instead, children may vary in how much they apply their mentalizing abilities for ambiguity resolution in the PI condition: those who mentalized the speakers’ intention succeeded, while those who did not may miss the correct mapping between word and its referent. The setup of the pragmatic-inference condition is always “I want this ___! It’s holding a ___!” with one figure holding a solo novel object, and the other holding two – one identical to the solo and one unique. While the indefinite article “a” can be interpreted as referring to a unique object as much as a solo object, prior experience with these interpretations may cue some children to place greater weight on “a” solo object, disregarding that this object is also often unique. Thus, when hearing a statement such as “a mel,” they may map “mel” to the solo object, selecting the incorrect figure. Alternatively, others may struggle with weighting the sematic vs. pragmatic cue and become inconsistent in selection. This cue weighting may not stem fully from semantic vs. pragmatic domain-weighting, but from observed variability in local vs. global processing in autistic individuals. Past research has found variability in preferences for or enhancements in local processing over global in autistic individuals (Plaisted et al., 1999; Frith and Happé, 1994; Koldewyn et al., 2013; Behrmann et al., 2006; Hayward et al., 2012). As such, the semantic cue of “a,” which is in local proximity to the novel word and involves less integration and processing of the full global linguistic, pragmatic, and visual context, may be more salient for some autistic individuals, leading to greater chances of incorrect mapping. This is further reinforced by the behavioral contrasts with our neurotypical individuals. The autistic PI-Limited group shows little evidence of successful meaning mapping in the PI condition. In contrast, both the neurotypical PI-Limited group matched in age and the younger group matched in mentalizing skills struggled in retaining the meanings, rather than in mapping them in the first place. However, future work will need to explore pragmatic inferencing in even-younger neurotypical children to better tease apart whether this indicates an underlying word-learning path different from non-autistic children or a developmental delay.

For retention, then, what may underpin the PI advantage after mapping success? Mentalizing seems like a strong candidate, with its replicated link in non-autistic individuals (Trice et al., 2023, 2024, 2025). This possibility is undercut by the lack of between-group differences in mentalizing metrics. However, here we can consider mentalizing as a trait—one’s baseline skill—vs. state—one’s current application. Our mentalizing tasks are coarse *trait* measures, and they probe circumstances where the need for mentalizing is very explicit or outsider observations equate to internal mentalizing abilities. However, we know that mentalizing *state* is malleable within-individual and correlates specifically with PI resolution and retention (Trice et al., 2024). In our word-learning scenarios, mentalizing is implicitly motivated. Thus, the group with neither correct resolution nor downstream retention boosts in PI may not be utilizing mentalizing as a state to resolve ambiguity. In contrast, the group with correct resolution and a memory-boosting effect for PI, like their typically-developing peers, may be utilizing mentalizing on the state level. Future work can probe this distinction both on a correlational level, by examining concurrent cognitive systems during mapping via neuroimaging, and a causational level, by perturbation of the targeted system through behavioral priming (e.g., Trice et al., 2024). This would allow us to determine whether mentalizing *state* or other cognitive mechanisms results in different sound-meaning mapping outcomes in the PI context.

The need for further research in mentalizing state or other cognitive mechanisms is underpinned by our eye-tracking results. In the group succeeding at PI mapping and retention, a delay in resolution relative to their neurotypical peers was still seen. The lack of individuating measures makes it difficult to determine whether this delay stems from qualitatively-different or delayed-but-functionally-equivalent mechanisms. Critically, this late divergence did not undercut mapping or retention accuracy. Thus, it may not mark greater difficulty, but instead a compensatory strategy to minimize the possibility of early prediction error. Future work will need to further examine online cognitive processes and extend this work to non-pragmatic predictive learning scenarios to tease such possibilities apart.

When considering practical implications, the lack of group differentiation by mentalizing and language measures, individually or in-consort, is particularly salient. Instead, the combination of immediate mapping and non-verbal IQ seem to reliably predict who has the potential to benefit from mapping words via PI. These findings underscore the importance of characterizing learning process when evaluating children’s mental readiness for certain type of word learning. When ambiguity is present, a significant subgroup of autistic children are at-risk of drawing an atypical and unintended or incorrect conclusion as to the intended referent. However, these aren’t always the individuals that teachers would view as needing additional language or social communication support. Thus, while these children may have opportunities to resolve incorrectly-mapped words at later occurrences in naturalistic settings, allowing them to remain on-par with peers in broadly-measured linguistic ability, this does not prevent all potential impacts, from additional cognitive resources necessitated to later correct mapping to confounds in the comprehension of the incorrectly-mapped word to the additional social and emotional pressures of making linguistic errors in a group already at-risk for increased rates of bullying, anxiety, depression, and low self-esteem (Accardo et al., 2024; van der Cruijsen and Boyer, 2021).

When considering stimuli, several possible confounds exist. Perceptually, the pragmatic-inference condition has an additional novel object—the competitor—which may elicit interest and increase attention. While Trice et al. (2025) has demonstrated that this is unlikely to be the primary factor in neurotypical children, further work in autism examining the impact of competitors in non-pragmatic ambiguity is needed. Linguistically, however, it is direct mapping that may have increased saliency and attention to its novel object, as it is the target of the selection as opposed to a clarifying feature. While better memory of pragmatically-inferred words (when correctly mapped) weighs against this, future work should still explore the impact of linguistic features. Finally, while word-object pairs were assigned at-random, the combinations for one condition may be more memorable. While the significant role of pragmatic-inference mapping success in any memory advantage minimizes the likelihood of this as the driving factor, replications should recombine stimuli.

When considering generalizability, the autistic children sampled here have typical-like verbal abilities and non-verbal IQ. Thus, our findings may not hold for those autistic children most at-risk of language disorders or with intellectual disability. Studying word learning in such populations poses significant methodological challenges in capturing and interpreting behavior. However, non-verbal and low-demand metrics like eye-gaze via home webcams succeeded in teasing apart our sub-groups. Thus, our experimental paradigm has the potential to be generalized to a wider range of the autistic population. An additional consideration is the binary nature of our testing – either the underlying assumptions, designed to be rooted in informativity, were correctly applied in each instance of a specific framework, or they were not. Thus, it is critical that future work expand this to pragmatic inferences utilizing other pragmatic computations guided by alternative principles. This will reveal the degree to which sub-group membership is consistent across varying pragmatic skills within-individual.

## Data Availability

The datasets presented in this study can be found in online repositories. The names of the repository/repositories and accession number(s) can be found at: https://osf.io/w79j6/?view_only=d944bdb8d3cb4924931db9af8637e8f4.

## Footnotes

^1^Two trials in the PI condition and three trials in the DM condition were removed from the analyses, due to invalid button presses within 100 ms after visual stimuli appeared on the screen, resulting in 3 instead of 4 test trials for those children.
