# Supplementary material for: From encoding to remembering: pragmatic inferences reveal distinct routes of word learning in autistic children
Source: Front Hum Neurosci. 2025 Aug 21;19:1633013. doi: 10.3389/fnhum.2025.1633013 (PMC12410077; doi:10.3389/fnhum.2025.1633013)
Supplement: Supplementary file 1 [file Data_Sheet_1.docx]

Supplementary Material

# Supplementary Tables

**Table A1. Further Participant Demographics**

| Demographic | Count | % |
| --- | --- | --- |
| Language Disorder Diagnosis |  |  |
| Yes | 26 | 53% |
| No | 23 | 47% |
| Neurological Disorder Diagnosis |  |  |
| Yes | 16 | 33% |
| No | 32 | 65% |
| Unknown | 1 | 2% |
| Psychological Disorder Diagnosis |  |  |
| Yes | 26 | 53% |
| No | 22 | 45% |
| Unknown | 1 | 2% |
| Genetic Disorder Diagnosis |  |  |
| Yes | 0 | 0 |
| No | 48 | 98% |
| Unknown | 1 | 2% |
| Past Traumatic Brain Injury |  |  |
| Yes | 1 | 2% |
| No | 47 | 96% |
| Unknown | 1 | 2% |
| Past Ear Infection with Some Level of Hearing Loss |  |  |
| Yes | 3 | 6% |
| No | 45 | 92% |
| Unknown | 1 | 2% |
| ASD Services |  |  |
| Yes | 33 | 67% |
| No | 13 | 27% |
| Unknown | 3 | 6% |
| IEP Services |  |  |
| Yes, Including Language | 18 | 37% |
| Yes, Excluding Language | 13 | 27% |
| No | 15 | 31% |
| Unknown | 3 | 6% |

*Note:* ASD, Autism Spectrum Disorder; IEP, Individualized Education Plan.

**Table A2. Individual Difference Measures for Full Group**

| Category | Demographic | Mean | SD | Count | % |
| --- | --- | --- | --- | --- | --- |
| Language | PVT Standard Score | 103.42 | 18.54 |  |  |
|  | ORR Standard Score | 106.47 | 16.28 |  |  |
|  | RSR Standard Score | 87.48 | 23.04 |  |  |
|  | SCQ Communication Sub-Score | 5.89 | 2.39 |  |  |
| Theory of Mind | Selected ToM Booklet Score* | 0.66 | 0.20 |  |  |
|  | MitE Socre | 8.38 | 2.37 |  |  |
|  | ABI-S Social Communication Score | 5.15 | 2.78 |  |  |
| Services | ASD Services** |  |  |  |  |
|  | Yes |  |  | 33 | 72% |
|  | No |  |  | 13 | 28% |
|  | IEP Services** |  |  |  |  |
|  | Yes, Including Language |  |  | 18 | 39% |
|  | Yes, Excluding Language |  |  | 13 | 28% |
|  | No |  |  | 15 | 33% |
| General | KBIT Score | 108.09 | 27.31 |  |  |
|  | Gender |  |  |  |  |
|  | Boy |  |  | 35 | 71% |
|  | Girl |  |  | 14 | 65% |
|  | Current Age (Years) | 7.58 | 0.88 |  |  |
|  | Diagnosis Age (Years) | 3.47 | 1.07 |  |  |

*Note that the Selected ToM Booklet Score excludes scores from the two subjects who got less that 2/3 of the ToM control questions incorrect

**Subjects who did not report whether or not their child received services are excluded in this count and percentage

*Note:* PVT, National Institute of Health Toolbox Picture Vocabulary Test (Standard Score); ORR, National Institute of Health Toolbox Oral Reading Recognition Test (Standard Score); RSR, Redmond Sentence Recall (Standard Score); SCQ, Social Communication Questionnaire; ToM, Theory of Mind; MitE, Mind in the Eyes. ABI-S, Autism Behavioral Inventory – Short; ASD, Autism Spectrum Disorder; IEP, Individualized Education Plan; KBIT, Kaufman Brief Intelligence Test-II Nonverbal Subtest (Standard Score).

**Table A3. Model Parameters for Immediate Recall and Retention for Full Sample**

| Effects | Immediate Recall | | | Retention | | |
| --- | --- | --- | --- | --- | --- | --- |
|  | Estimate | SE | *z* | Estimate | SE | *z* |
| Intercept | -0.30 | 0.26 | -1.12 | -0.35 | 0.16 | -2.17 * |
| Condition (Inference vs. Direct Mapping) | 0.48 | 0.48 | 1.00 | 0.89 | 0.30 | 2.92 ** |
| Gender | 0.68 | 0.42 | 1.60 | 0.22 | 0.30 | 0.74 |
| Condition x Gender | -0.50 | 0.72 | -0.70 | -0.54 | 0.52 | -1.03 |
| Previously Learned | 0.01 | 0.33 | 0.03 | 1.06 | 0.30 | 3.58 *** |

*Note:* Significance levels: . p < .1, * p < .05, ** p < .01, *** p < .001.

**Table A4. Individual Difference Measure by Subgroup**

|  |  | PI Retained | | | | | PI Limited | | | |  |
| --- | --- | --- | --- | --- | --- | --- | --- | --- | --- | --- | --- |
| Category | Demographic | Mean | SD | Count | | % | Mean | SD | Count | % | *p* |
| Language | PVT Standard Score | 102.12 | 16.56 | |  |  | 105.16 | 21.65 |  |  | > 0.1 |
|  | ORR Standard Score | 106.44 | 14.38 | |  |  | 105.22 | 18.55 |  |  | > 0.1 |
|  | RSR Standard Score | 88.29 | 23.31 | |  |  | 84.53 | 23.08 |  |  | > 0.1 |
|  | SCQ Communication Sub-Score | 5.17 | 2.36 | |  |  | 6.72 | 2.21 |  |  | = 0.05 |
| Theory of Mind | Selected ToM Booklet Score* | 0.70 | 0.19 | |  |  | 0.60 | 0.22 |  |  | > 0.1 |
|  | MitE Socre | 8.48 | 2.27 | |  |  | 8.28 | 2.61 |  |  | > 0.1 |
|  | ABI-S Social Communication Score | 4.77 | 2.45 | |  |  | 5.50 | 3.20 |  |  | > 0.1 |
| Services | ASD Services** |  |  | |  |  |  |  |  |  | > 0.1 |
|  | Yes |  |  | | 19 | 73.08 |  |  | 14 | 73.68 |  |
|  | No |  |  | | 7 | 26.92 |  |  | 5 | 26.32 |  |
|  | IEP Services** |  |  | |  |  |  |  |  |  | > 0.1 |
|  | Yes, Including Language |  |  | | 9 | 34.62 |  |  | 9 | 47.37 |  |
|  | Yes, Excluding Language |  |  | | 8 | 30.77 |  |  | 4 | 21.05 |  |
|  | No |  |  | | 9 | 34.62 |  |  | 6 | 31.58 |  |
| General | KBIT Score | 111.08 | 27.16 | |  |  | 102.47 | 27.33 |  |  | > 0.1 |
|  | Gender |  |  | |  |  |  |  |  |  | > 0.1 |
|  | Boy |  |  | | 18 | 66.67 |  |  | 16 | 76.19 |  |
|  | Girl |  |  | | 9 | 33.33 |  |  | 5 | 23.81 |  |
|  | Current Age (Years) | 7.51 | 0.83 | |  |  | 7.66 | 0.95 |  |  | > 0.1 |
|  | Diagnosis Age (Years) | 3.56 | 1.06 | |  |  | 3.48 | 0.99 |  |  | > 0.1 |

*Note that the Selected ToM Booklet Score excludes scores from the two subjects who got less that 2/3 of the ToM control questions correct

**Subjects who did not report whether or not their child received services are excluded in this count and percentage

*Note:* PVT, National Institute of Health Toolbox Picture Vocabulary Test (Standard Score); ORR, National Institute of Health Toolbox Oral Reading Recognition Test (Standard Score); RSR, Redmond Sentence Recall (Standard Score); SCQ, Social Communication Questionnaire; ToM, Theory of Mind; MitE, Mind in the Eyes. ABI-S, Autism Behavioral Inventory – Short; ASD, Autism Spectrum Disorder; IEP, Individualized Education Plan; KBIT, Kaufman Brief Intelligence Test-II Nonverbal Subtest (Standard Score).

**Table A5. Model Parameters for Cumulative Individual Difference Models for Group Membership**

| Effects | Estimate | SE | *z* |
| --- | --- | --- | --- |
| PI Initial Mapping Alone |  |  |  |
| Intercept | -2.01 | 0.81 | -2.47 * |
| **Initial PI Mapping** | 3.72 | 1.22 | 13.05 ** |
| PI Initial Mapping + Age |  |  |  |
| Intercept | -1.68 | 3.15 | -0.54 |
| **PI Initial Mapping** | 3.70 | 1.23 | 3.01 ** |
| Age (Years) | -0.04 | 0.39 | -0.11 |
| PI Initial Mapping + KBIT |  |  |  |
| Intercept | -8.06 | 2.86 | -2.82 ** |
| **PI Initial Mapping** | 6.17 | 1.82 | 3.39 *** |
| **KBIT (Standard Score)** | 0.04 | 0.02 | 2.34 ** |
| KBIT Alone |  |  |  |
| Intercept | -1.10 | 1.20 | -0.92 |
| KBIT (Standard Score) | 0.01 | 0.01 | 1.26 |
| PI Initial Mapping + KBIT + Mentalizing |  |  |  |
| Intercept | -7.80 | 2.96 | -2.63 ** |
| **PI Initial Mapping** | 6.11 | 1.84 | 3.33 *** |
| **KBIT (Standard Score)** | 0.04 | 0.02 | 2.12 * |
| Mentalizing | 0.40 | 0.64 | 0.54 |
| PI Initial Mapping + KBIT + Language |  |  |  |
| Intercept | -8.81 | 3.11 | -2.84 ** |
| **PI Initial Mapping** | 6.58 | 1.97 | 3.33 *** |
| **KBIT (Standard Score)** | 0.05 | 0.02 | 2.43 * |
| Language | -0.59 | 0.73 | -0.80 |

*Note:* Bolded text represents significant individual difference measures. PI, Pragmatic Inference; KBIT, Non-Verbal Kaufman Brief Intelligence Test-II. Significance levels: . p < .1, * p < .05, ** p < .01, *** p < .001.

**Table A6. Cross-Validation Results**

| Category | Variables | Co-Efficient |
| --- | --- | --- |
|  | Intercept | 0.30 |
| Language | PVT Standard Score | 0.00 |
|  | ORR Standard Score | 0.00 |
|  | RSR Standard Score | 0.00 |
|  | SCQ Communication Sub-Score | -0.00 |
| Theory of Mind | Selected ToM Booklet Score | 0.00 |
|  | MitE Socre | 0.00 |
|  | ABI-S Social Communication Score | 0.00 |
| Services | ASD Services (Yes/No) | 0.00 |
|  | IEP Services (Yes, Including Language/Yes, Excluding Language/No) | 0.00 |
| General | KBIT Score | 0.00 |
|  | Gender (Boy/Girl) | 0.00 |
|  | Current Age (Years) | 0.00 |
|  | Diagnosis Age (Years) | 0.00 |

*Note:* PVT, National Institute of Health Toolbox Picture Vocabulary Test (Standard Score); ORR, National Institute of Health Toolbox Oral Reading Recognition Test (Standard Score); RSR, Redmond Sentence Recall (Standard Score); SCQ, Social Communication Questionnaire; ToM, Theory of Mind; MitE, Mind in the Eyes. ABI-S, Autism Behavioral Inventory – Short; ASD, Autism Spectrum Disorder; IEP, Individualized Education Plan; KBIT, Kaufman Brief Intelligence Test-II Nonverbal Subtest (Standard Score).

# Supplementary Figures


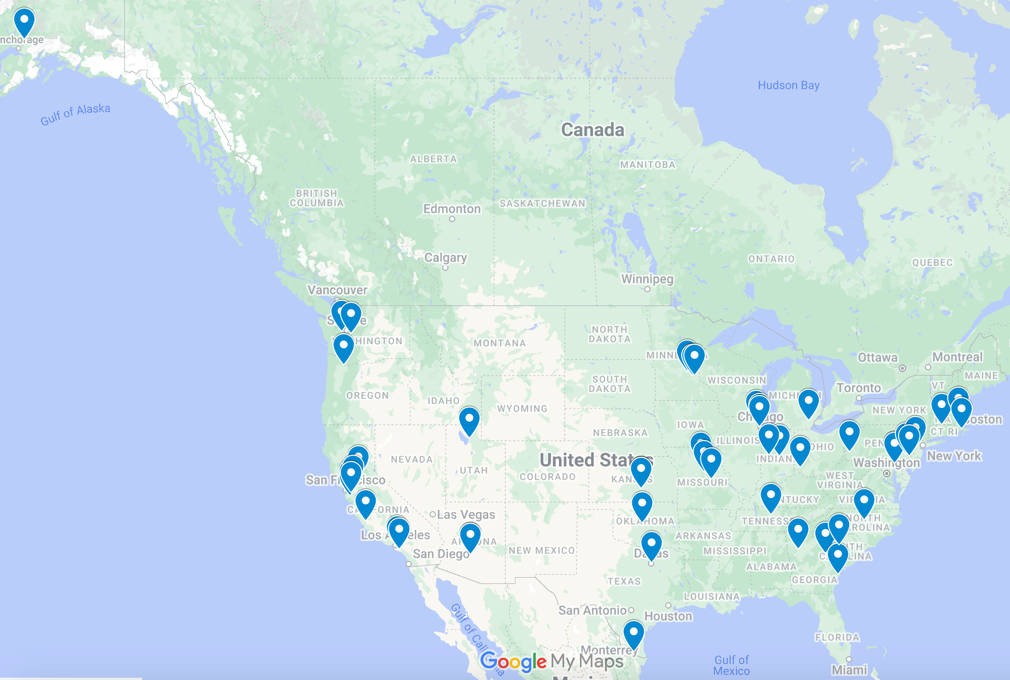


**Figure A1. Map of Geographic Locations of Participants.** Note that not all participant’s locations were able to be identified.

# Supplementary Descriptions of Individual Difference Measures

## Theory of Mind (ToM) Booklet Task

The ToM Booklet Task tests an array of conventional ToM constructs and was administered via Powerpoint in the 15 minutes between the final block of word learning and retention. This was a modified version of the task developed by Richardson et al, 2018 – a booklet that worked through, in our case, 11 interactive stories that all probed children’s theory of mind. This interactive assessment covered a wide variety of theory of mind constructs that develop across our age range, ranging from multiple complexities of false belief to moral reasoning to diverse desires, allowing us to obtain a more holistic view of theory of mind variability (Cushman et al., 2013; Wellman et al., 2001).

As this was a highly verbal task, control questions which relied solely on language comprehension and recall for referential disambiguation were included to capture baseline. Thus a total of 26 mentalizing questions, 4 generic comprehension questions (of which three required participants to chose between referents and were thus our control questions), and several unscored questions used for set up and transition within stories were asked across all 11 stories. Participants who answered more than 1 of the control questions incorrectly had their ToM booklet data excluded from later analyses under the concern that their performance on the ToM task may reflect difficulties in language comprehension or referential disambiguation rather than ToM itself. Thus, 2 subjects had this data excluded.

Because of the subjective nature of this task, children’s responses were double-scored by trained research assistants from the session recording. If these scores differed on any given question and the scorers could not reach consensus, a third coder (the first author) would watch the video and determine the final score. This task is identical to the one used in Trice et al, 2025.

## Mind in the Eyes, Child Version

The Mind in the Eyes Task evaluates emotional identification of facial expressions and was administered via Gorilla and consisted of 14 trials in randomized order. Each consisted of one photograph of a child’s eyes and four accompanying words, of which one correctly matched the emotion captured in the photograph. A brief introduction introduced the conceit of the task – that children should choose the word that best describes what the person in the picture is thinking or feeling – along with a practice trial to verify understanding.

Because of the varying reading skills of the participant, for each trial, the experimenter would ask what the person in the picture was feeling and read off all four options. They would then select the option the participant chose. While this task was designed to have child-appropriate emotions and language, children were also instructed to ask the experimenter the definition of any word that they did not know to limit impacts of vocabulary knowledge discrepancies.

# Supplementary Descriptions of Analysis Approach and Models

## Immediate Recall, Retention, and Generalized Linear Mixed Effects Modeling

Generalized binomial linear mixed models (*glmer*) were constructed to separately analyze immediate recall and retention using the lme4 package of R (Baayen, 2008; Baayen et al., 2008). This allowed us to control for a) possible effects of initial mapping accuracy and gender and b) for individual participant variability in memory while comparing PI and DM conditions. In each case, trial-by-trial binomial accuracy was coded as 1 for correct and 0 for incorrect. An additional variable marking whether or not a given word was learned was coded based on initial mapping accuracy across its two learning trials – 1 for learned if both were correct, 0 for not learned in all other cases. In each model, the trial-by-trial accuracy for the given timepoint served as the dependent variable, with a central main effect of condition. Subsequently, the interaction of gender with condition was included, and the learned variable served as a covariate. For our qualitative variables, contrast coding of -0.5 and 0.5 were used. By-subject random intercepts and random slopes for condition and by-item random intercepts were included.

## Eye-tracking Analyses

A divergence-point analysis was used to capture the timeline of referential disambiguation, examining when proportion of looks-to-the-target diverged from looks-to-the-competitor an whether this significantly differed between groups. Here, a moving window of 33 ms was applied to allow for about 64 observations per bin for a given participant. The subsequent analysis made us of the R eye-tracking analysis package stats.VWP (Ito & Knoeferle, 2022). This analysis was bootstrapped 1000 times, with the log of the fixation proportion for the given time bin per participant per trial calculated for the target and competitor for each. In each case, a t-test examined is this differed significantly for the given sub-group at a given timepoint. The divergence point was set where ten consecutive time bins were significantly different. This allowed us to ensure that the first and final bin had independent observations despite the moving window. 95% confidence intervals for the mean divergence point for each sub-group were calculated. If a divergence point was found for each group, the data would be pooled and randomly assigned labels for another 1000 iterations of our bootstrapping, forming a null distribution. A p-value would be calculated based on the null distribution proportion of occurrences larger than the differences in divergence points from the subgroups.

## Individual Difference Measures and Subgroup Membership

Note that our full group was split into two subgroups. This was re-enforced through the use of Gaussian mixture modeling *(mclust,* Scrucca et al., 2023). The analysis methods (beyond t-tests) to determine if any specific individual difference measures uniquely predict sub-groups membership are described below.

Our first approach was theoretically-motivated. As such, generalized linear models predicted subgroup membership with initial PI mapping accuracy as a baseline and nonverbal IQ and age as covariables. Our main variable of interest was either a composite score of language metrics, stemming from the fact that this study investigates language learning, or a composite score of mentalizing metrics, stemming from the link in other populations, as reported in Trice et al (2025) and Trice et al (2023), to this construct. In each case, the composite score for each child was computed via normalizing each of the individual difference measures in each domain (e.g., language and mentalizing) then averaging across these measures. We tested these models in a stepwise manner by incrementally adding predictors. The McFadden R^2^ was calculated when a model achieved significant prediction via the *pscl* package in R (Long, 1997).

Since we had many other variables of interest, a data driven cross-validation approach was used to determine if any of our other individual difference measures could tease apart our sub-group, with the exclusion of initial mapping as that was explored in earlier analyses. Here, as some subjects were missing data for individual difference measures because of task tolerance for a given subject on a given day, we used R’s *mice* package to generate multiple imputations (replacement values) for said missing data (van Buuren & Groothuis-Oudshoorn, 2011). Five possible data-frames were generated and used in a six-fold cross-validated stacked adaptive elastic net via R’s *miselect* package (Du et al., 2022) and its coefficients extracted.

# Supplementary Material Bibliography

Baayen, R.H. (2008). Analyzing linguistic data: a practical introduction to statistics using R, 1st Edition.

Baayen, R. H., Davidson, D. J., & Bates, D. M. (2008). Mixed-effects modeling with crossed random effects for subjects and items. *Journal of Memory and Language, 59*(4), 390–412. [https://doi.org/10.1016/j.jml.2007.12.005](https://psycnet.apa.org/doi/10.1016/j.jml.2007.12.005)

Cushman, F., Sheketoff, R., Wharton, S., & Carey, S. (2013). The development of intent-based moral judgment. *Cognition*, *127*(1), 6–21. <https://doi.org/10.1016/j.cognition.2012.11.008>

Du, J., Boss, J., Han, P., Beesley, L. J., Kleinsasser, M., Goutman, S. A., … Mukherjee, B. (2022). Variable Selection with Multiply-Imputed Datasets: Choosing Between Stacked and Grouped Methods. *Journal of Computational and Graphical Statistics, 31*(4), 1063–1075. https://doi.org/10.1080/10618600.2022.2035739

Ito, A., and Knoeferle, P. (2022). Analysing data from the psycholinguistic visual-world paradigm: comparison of different analysis methods. *Behav. Res. Methods* 55, 3461–3493. https://doi.org/10.3758/s13428-022-01969-3

Long, J.S. (1997). *Regression Models for Categorical and Limited Dependent Variables*. Thousand Oaks, California: Sage.

Richardson, H. et. al. (2018). Development of the social brain from age three to twelve years. *Nature Communications, 9*, 1027. <https://doi.org/10.1038/s41467-018-03399-2>

Scrucca L., Fraley C., Murphy T. B. and Raftery A. E. (2023) Model-Based Clustering, Classification, and Density Estimation Using mclust in R. Chapman & Hall/CRC, ISBN: 978-1032234953, <https://mclust-org.github.io/book/>

Trice, K. M., Saratsli, D., Papafragou, A., & Qi, Z. (2023). Pragmatics and social cognition in learning and remembering words. <https://doi.org/10.31234/osf.io/gv4an>

Trice K., Saratsli D., Papafragou A., & Qi Z. (2025). [The unforgettable “mel”: Pragmatic inferences affect how children acquire and remember word meanings.](https://onlinelibrary.wiley.com/doi/10.1111/desc.70013) Developmental Science. 28(3): e70013. DOI: 10.1111/desc.70013.

van Buuren, S., & Groothuis-Oudshoorn, K. (2011). mice: Multivariate Imputation by Chained Equations in R. *Journal of Statistical Software*, *45*(3), 1–67. <https://doi.org/10.18637/jss.v045.i03>

Wellman, H. M., Cross, D., & Watson, J. (2001). Meta-analysis of theory-of-mind development: The truth about false belief. *Child Development*, 72, 655–684. <https://doi.org/10.1111/1467-8624.00304>
